# Supplementary material for: Identification of photoperiod-induced specific miRNAs in the adrenal glands of Sunite sheep (Ovis aries)
Source: Front Vet Sci. 2022 Jul 22;9:888207. doi: 10.3389/fvets.2022.888207 (PMC9354845; doi:10.3389/fvets.2022.888207)
Supplement: Supplementary file 1 [file Data_Sheet_1.ZIP › Supplementary file/miRDeep2 file.zip!/miRDeep2 file/Know_Structure/SP42A1/oar-mir-103.pdf]

miRBase precursor	: oar-mir-103
Total read count	: 14743
oar-miR-103 read count	: 14743
remaining reads	: 0

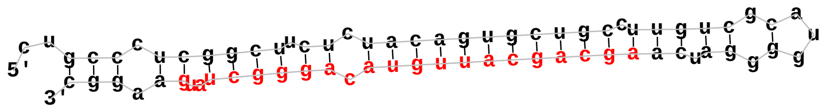

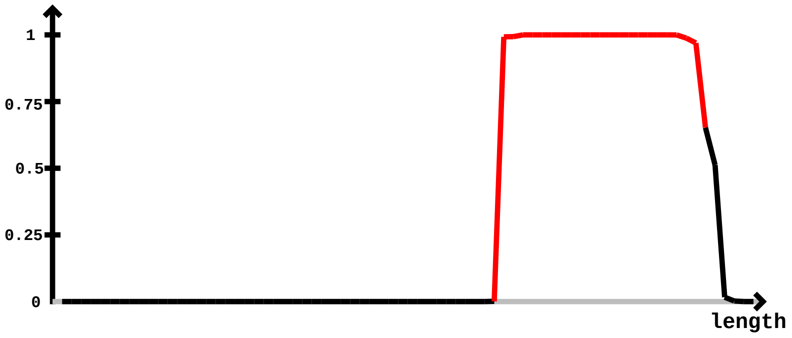


```
                                             oar-miR-103
5'- cugcccucggcuucucuacagugcugccuugucgcauggggaucaagcagcauuguacagggcuaugaaggc   -3'    exp
    ..(((.((((((.((.(((((((((((.(((((.(...).)).))))))))))))))).))))))..)).)))   reads      mm         sample
    ............................................aagcagcauuguacagggcuau......   3          0          seq
    ............................................aagcagcauuguacagggcuaug.....   5          0          seq
    ............................................aagcagcauuguacagggcuauga....   6          0          seq
    .............................................agcagcauuguacagggc.........   4          0          seq
    .............................................agcagcauuguacagggcu........   174        0          seq
    .............................................agcagcauuguacagggcua.......   267        0          seq
    .............................................agcagcauuguacagggcuau......   4675       0          seq
    .............................................agcagcauuguacagggcuaug.....   2050       0          seq
    .............................................agcagcauuguacagggcuauga....   7216       0          seq
    .............................................agcagcauuguacagggcuaugaa...   208        0          seq
    .............................................agcagcauuguacagggcuaugaag..   1          0          seq
    .............................................agcagcauuguacagggcuaugaaA..   26         1          seq
    ..............................................gcagcauuguacagggcuau......   3          0          seq
    ..............................................gcagcauuguacagggcuaug.....   1          0          seq
    ..............................................gcagcauuguacagggcuauga....   7          0          seq
    ...............................................cagcauuguacagggcu........   1          0          seq
    ...............................................cagcauuguacagggcuau......   1          0          seq
    ...............................................cagcauuguacagggcuaug.....   11         0          seq
    ...............................................cagcauuguacagggcuauga....   78         0          seq
    ...............................................cagcauuguacagggcuaugaa...   2          0          seq
    ...............................................cagcauuguacagggcuaugaag..   1          0          seq
    ...............................................cagcauuguacagggcuaugaaA..   3          1          seq
```
